# ﻿Morphological characteristics and phylogenetic evidence reveal two new species of *Acremonium* (Hypocreales, Sordariomycetes)

**DOI:** 10.3897/mycokeys.91.86257

**Published:** 2022-07-15

**Authors:** Xin Li, Zhi-Yuan Zhang, Yu-Lian Ren, Wan-Hao Chen, Jian-Dong Liang, Ji-Mei Pan, Jian-Zhong Huang, Zong-Qi Liang, Yan-Feng Han

**Affiliations:** 1 Institute of Fungus Resources, Department of Ecology, College of Life Sciences, Guizhou University, Guiyang 550025, Guizhou, China Guizhou University Guiyang China; 2 Basic Medical School, Guizhou University of Traditional Chinese Medicine, Guiyang 550025, Guizhou, China Guizhou University of Traditional Chinese Medicine Guiyang China; 3 Engineering Research Center of Industrial Microbiology, Ministry of Education, Fujian Normal University, Fuzhou 350108, Fujian, China Fujian Normal University Fuzhou China

**Keywords:** *Acremonium*, filamentous fungi, phylogeny, taxonomy

## Abstract

Using chicken feathers as bait, *Acremoniumglobosisporum***sp. nov.** and *Acremoniumcurvum***sp. nov.** were collected from the soil of Yuncheng East Garden Wildlife Zoo and Zhengzhou Zoo in China. They were identified by combining the morphological characteristics and the two-locus DNA sequence (LSU and ITS) analyses. In the phylogenetic tree, both new species clustered into separate subclades, respectively. They were different from their allied species in their morphology. The description, illustrations, and phylogenetic tree of the two new species were provided.

## ﻿Introduction

The genus *Acremonium* Link, established in 1929, with *A.alternatum* Link as the type species, is one of the largest and most complex genera of asexually typified. The morphological characteristics consist of hyphae septate, mostly tapered and lateral phialides, produced singly or in small groups, and unicellular conidia produced in mucoid heads or unconnected chains (Summerbell et al. 2011). Nowadays, *Acremonium* has 217 records in the Index Fungorum (http://www.indexfungorum.org/Names/Names.asp, retrieval on 30 Jun. 2022). The traditionally circumscribed *Acremonium* is polyphyletic, which explains why many *Acremonium* species were transferred to other genera and families (Yang et al. 2019). Thus, there are still many unidentified, suspect or misidentified taxa that require taxonomic investigation.

Due to the poor differentiation of asexual forms of the genus *Acremonium*, it is difficult to identify species only by morphological differences. To address this issue, there are many unidentified and suspicious species that require further phylogenetic analysis. To date, many isolates of *Acremonium* spp. lack the gene loci such as SSU, *TEF 1-α* and *RPB2* (Table 1), therefore, phylogenetic analyses of this genus are generally performed based on the single locus sequences, especially LSU (Hyde et al. 2020).

In the present study, two new species of *Acremonium* were identified in a survey of keratinolytic fungi from China, which were enriched by the baiting technique. We provided a description, illustrations, and phylogenetic tree for the two new species.

## ﻿Materials and methods

### ﻿Fungal isolation and morphology

Soil samples were collected from Yuncheng East Garden Wildlife Zoo (35°6'26"N, 111°4'24"E) (three isolates), Yuncheng City, Shanxi Province and Zhengzhou Zoo (34°47'20"N, 113°40'41"E) (one isolate), Zhengzhou City, Henan Province, China by Yu-Lian Ren on July 2021. We collected 3–10 cm below the soil surface, placed the samples in sterile Ziploc plastic bags (Kaixin Biotechnology, Guizhou, China), and transported them to the laboratory (Zhang et al. 2019a, b). Then, they were treated and isolated according to the baiting method (using chicken feathers as bait: a method specifically designed for isolating keratinophilic microbes) of Zhang et al. (2020a, b; 2021). We washed the chicken feathers, sterilized them in an autoclave for 30 minutes at 121 °C, and dried them in an oven at 50 °C. The sterile and dried chicken feathers were mixed with soil samples and then wet with sterile distilled water and cultured at darkroom temperature for 1 month (Li et al. 2022).

Then, the 2 g samples were weighed in a conical flask with glass beads containing 20 mL sterile water and mixed evenly by eddy shock for 10 min. Next, 1 mL samples were mixed evenly in 9 mL sterile water in a sterile environment and diluted to 10^-3^. Then, 1 mL 10^-3^ samples were put into a sterile petri dish, and SDA medium containing 50 mg/L penicillin and 50 mg/L streptomycin was added and mixed. The target strains were isolated. The purified strains were transferred to PDA, OA, and MEA plates for dark culture at 25 °C for 7 days. Microscopic features were examined by making direct wet mounts with 25% lactic acid on PDA, with a light microscope.

The cultures were placed to slowly dry at 50 °C to produce the dried holotype. The dried holotype was deposited in the Mycological Herbarium of the Institute of Microbiology, Chinese Academy of Sciences, Beijing, China (**HMAS**), while ex-type living culture was stored in PDA test tubes which were deposited in the China General Microbiological Culture Collection Center (**CGMCC**), and the Institute of Fungus Resources, Guizhou University, Guiyang City, Guizhou, China (**GZUIFR**).

### ﻿DNA extraction, PCR amplification, and sequencing

We used a 5% chelex-100 solution for total genomic DNA extraction. ITS1/ITS4 (White et al. 1990), LROR/LR7 (Vilgalys and Hester 1990), EF1-983F/ EF1-2218R (Rehner and Buckley 2005), fRPB2-5f/ fRPB2-7cR (Liu et al. 1999), and NS1 and NS4 (White et al. 1990) primers were used for amplification of the internal transcribed spacers (ITS), the 28S nrRNA locus (LSU), translation elongation factor 1-alpha gene region (TEF 1-α), RNA polymerase II second largest subunit gene (RPB2), and small subunit rDNA (SSU), respectively. Purification and sequencing were performed by Quintarabio (Wuhan, China). The new sequences were submitted to GenBank (Table 1).

**Table 1.** Strains included in the present study.

Species	Strains	LSU	ITS	SSU	*TEF 1*-α	*RPB2*
*Acremoniumalcalophilum*	CBS 114.92^T^	JX158443	DQ825967	JX158486	JX158399	JX158465
*Acremoniumalternatum*	CBS 407.66^T^	HQ231988	HE798150			
*Acremoniumalternatum*	CBS 831.97	HQ231989				
*Acremoniumarthrinii*	MFLU 18-1225^T^	MN036334		MN036335	MN038169	
*Acremoniumbehniae*	CBS 146824^T^	MW175400	MW175360			
*Acremoniumbiseptum*	CBS 750.69^T^	HQ231998				
*Acremoniumblochii*	CBS 993.69	HQ232002	HE608636			
*Acremoniumborodinense*	CBS 101148^T^	HQ232003	HE608635			
*Acremoniumbrachypenium*	CBS 866.73^T^	HQ232004	AB540570			
*Acremoniumcamptosporum*	CBS 756.69^T^	HQ232008		HQ232186		
*Acremoniumcavaraeanum*	CBS 101149^T^	HF680202	HF680220			
*Acremoniumcavaraeanum*	CBS 111656	HF680203	HF680221			
*Acremoniumcavaraeanum*	CBS 758.69	HQ232012	HF680222			
*Acremoniumcerealis*	CBS 207.65	HQ232013				
*Acremoniumcerealis*	CBS 215.69	HQ232014				
*Acremoniumchiangraiense*	MFLUCC 14-0397^T^	MN648329	MN648324			
*Acremoniumchrysogenum*	CBS 144.62^T^	HQ232017		HQ232187		
*Acremoniumchrysogenum*	CBS 401.65	MH870276	MH858636			
*Acremoniumcitrinum*	CBS 384.96^T^	HF680217	HF680236			
*Acremoniumdimorphosporum*	CBS 139050^T^	LN810506	LN810515			
*Acremoniumexiguum*	CBS 587.73^T^	HQ232035				
*Acremoniumexuviarum*	UAMH 9995^T^	HQ232036	AY882946			
*Acremoniumfelinum*	CBS 147.81^T^	AB540488	AB540562			
*Acremoniumflavum*	CBS 596.70^T^	HQ232037		HQ232191		
*Acremoniumflavum*	CBS 316.72	MH872204	MH860487			
*Acremoniumfuci*	CBS 112868^T^		AY632653			
*Acremoniumfuci*	CBS 113889		AY632652			
*Acremoniumfusidioides*	CBS 109069	HF680204	HF680223			
*Acremoniumfusidioides*	CBS 991.69	HF680211	HF680230			
*Acremoniumfusidioides*	CBS 840.68^T^	HQ232039	FN706542			
*Acremoniumhansfordii*	CBS 390.73	HQ232043	AB540578			
*Acremoniumhennebertii*	CBS 768.69^T^	HQ232044	HF680238			
*Acremoniuminflatum*	CBS 212.69^T^	HQ232050				
*Acremoniummali*	ACCC 39305^T^	MF993114	MF987658			
*Acremoniummoniliforme*	CBS 139051^T^	LN810507	LN810516			
*Acremoniummoniliforme*	FMR 10363	LN810508	LN810517			
*Acremoniumparvum*	CBS 381.70A	HQ231986	HF680219			
*Acremoniumpersicinum*	CBS 310.59^T^	HQ232077				
*Acremoniumpersicinum*	CBS 101694	HQ232085				
*Acremoniumpinkertoniae*	CBS 157.70^T^	HQ232089		HQ232202		
*Acremoniumpolychroma*	CBS 181.27^T^	HQ232091	AB540567			
*Acremoniumpotronii*	CBS 189.70	HQ232094				
*Acremoniumpseudozeylanicum*	CBS 560.73^T^	HQ232101				
*Acremoniumpteridii*	CBS 782.69^T^	HQ232102				
*Acremoniumpteridii*	CBS 784.69	HQ232103				
*Acremoniumsclerotigenum*	CBS 124.42^T^	HQ232126	FN706552	HQ232209		
*Acremoniumsclerotigenum*	A101	KC987215	KC987139	KC987177	KC998961	
*Acremoniumsclerotigenum*	A130	KC987242	KC987166	KC987204	KC998988	
*Acremonium* sp.	E102	KC987248	KC987172	KC987210	KC998994	KC999030
*Acremoniumspinosum*	CBS 136.33^T^	HQ232137	HE608637	HQ232210		
*Acremoniumstroudii*	CBS 138820^T^		KM225291			
*Acremoniumtumulicola*	CBS 127532^T^	AB540478	AB540552			
*Acremoniumvariecolor*	CBS 130360^T^	HE608651	HE608647			
*Acremoniumvariecolor*	CBS 130361	HE608652	HE608648			
*Acremoniumverruculosum*	CBS 989.69^T^	HQ232150				
*Acrophialophorahechuanensis*	GZUIFR-H08-1^T^	MK926789	DQ185070	EU053286		
*Brunneomycesbrunnescens*	CBS 559.73^T^	HQ231966	LN810520	HQ232184	LN810534	
*Brunneomyceshominis*	UTHSC 06-415^T^	LN810509	KP131517		LN810535	
*Bryocentriabrongniartii*	M139	EU940105		EU940052		
*Bryocentriabrongniartii*	M190	EU940125		EU940052		
*Bryocentriametzgeriae*	M140	EU940106				
*Bulbitheciumhyalosporum*	CBS 318.91^T^	AF096187	HE608634			
*Cephalosporiumpurpurascens*	CBS 149.62^T^	HQ232071				
*Cosmosporalavitskiae*	CBS 530.68^T^	HQ231997				
*Emericellopsisalkalina*	CBS 127350^T^	KC987247	KC987171	KC987209	KC998993	KC999029
*Emericellopsisterricola*	CBS 120.40^T^	U57082	U57676	U44112		
*Gliomastixroseogrisea*	CBS 134.56^T^	HQ232121				
*Hapsidosporairregularis*	ATCC 22087^T^	AF096192		AF096177		
*Kiflimoniumcurvulum*	CBS 430.66^T^	HQ232026	HE608638	HQ232188		
*Lanatonectriaflavolanata*	CBS 230.31	HQ232157				
*Leucosphaerinaarxii*	CBS 737.84^T^	HE608662	HE608640			
*Nigrosabulumglobosum*	ATCC 22102^T^	AF096195				
*Paracremoniumcontagium*	CBS 110348^T^	HQ232118	KM231831		KM231966	
*Parasarocladiumbreve*	CBS 150.62^T^	HQ232005				
*Parasarocladiumradiatum*	CBS 142.62^T^	HQ232104		HQ232205		
*Pestalotiopsishawaiiensis*	CBS 114491^T^	KM116239	KM199339		KM199514	
*Pestalotiopsisspathulata*	CBS 356.86^T^	KM116236	KM199338		KM199513	
*Phialemoniumatrogriseum*	CBS 604.67^T^	HQ231981	HE610367	FJ176825		
*Pseudoacremoniumsacchari*	CBS 137990^T^	KJ869201	KJ869144			
*Sarcopodiumvanillae*	CBS 100582	HQ232174	KM231780		KM231911	
*Sarocladiumbacillisporum*	CBS 425.67^T^	HQ231992	HE608639	HQ232179		
*Sarocladiumbactrocephalum*	CBS 749.69^T^	HQ231994	HG965006	HQ232180		
*Sarocladiumstrictum*	CBS 346.70^T^	HQ232141	AY214439	HQ232211		
*Sarocladiumterricola*	CBS 243.59^T^	HQ232046		HQ232196		
*Seliniapulchra*	AR 2812	GQ505992	HM484859		HM484841	
*Trichotheciumcrotocinigenum*	CBS 129.64^T^	HQ232018	AJ621773			
*Trichotheciumindicum*	CBS 123.78^T^	AF096194		AF096179		
*Trichotheciumroseum*	DAOM 208997	U69891		U69892		
*Trichotheciumsympodiale*	ATCC 36477	U69889		U69890		
*Acremoniumcurvum*	CGMCC 3.20954 = GZUIFR 22.035^T^	ON041050	ON041034	ON876754	ON494579	ON494583
*Acremoniumglobosisporum*	CGMCC 3.20955 = GZUIFR 22.036^T^	ON041051	ON041035	ON876755	ON494580	ON494584
*Acremoniumglobosisporum*	GZUIFR 22.037	ON041052	ON041036	ON876756	ON494581	ON494585
*Acremoniumglobosisporum*	GZUIFR 22.038	ON041053	ON041037	ON876757	ON494582	ON494586

Notes: “^T^” stands for Ex-type strains.

### ﻿Phylogenetic analyses

The ITS and LSU sequences of *Acremonium* were downloaded from GenBank (Table 1). Two strains of *Pestalotiopsisspathulata* (CBS 356.86) and *P.hawaiiensis* (CBS 114491) were chosen as the outgroup taxa. The TBtools were used for name simplification and renaming (Chen et al. 2020). Sequences were aligned by MAFFT v7.037 (Katoh and Standley 2013). Multi-locus was concatenated by PhyloSuite v1.16 (Zhang et al. 2020a).

Bayesian inference (BI) and maximum likelihood (ML) methods were used in the analysis. For BI analysis was conducted with MrBayes v3.2 (Ronquist et al. 2012) and Markov chain Monte Carlo (MCMC) simulations; ML analysis was performed using IQ-TREE v1.6.11 (Nguyen et al. 2015), as outlined in Li et al (2022). All analyses were performed in PhyloSuite V1.16 (Zhang et al. 2020b).

## ﻿Results

### ﻿Phylogeny

**Figure 1.**
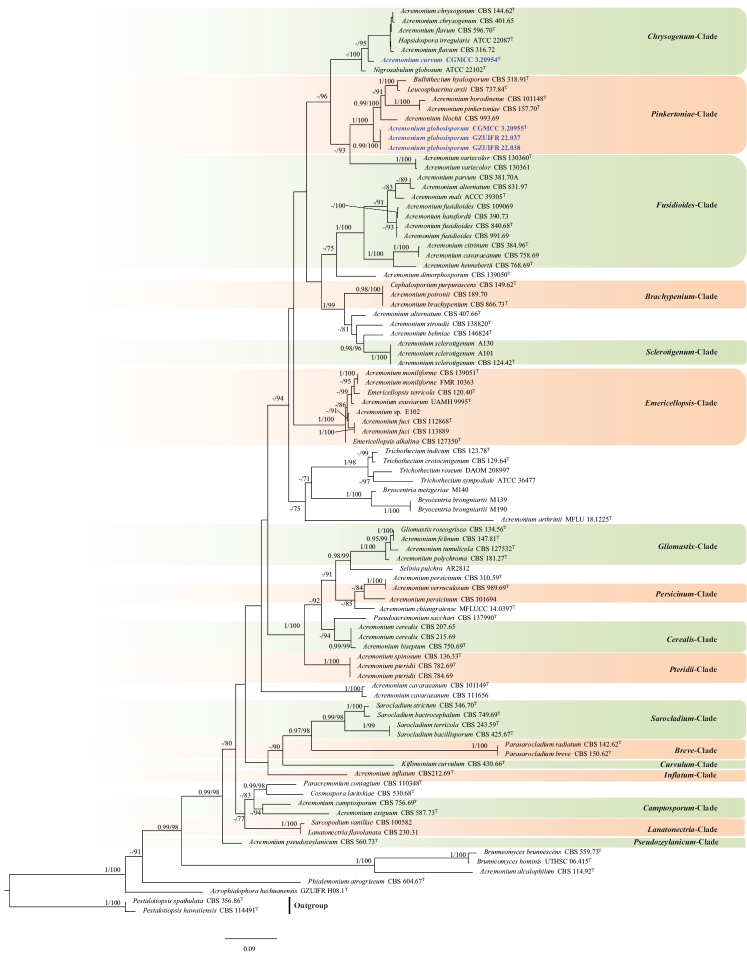
Phylogenetic tree of the genus *Acremonium* constructed from LSU and ITS. Bayesian posterior probability (≥ 0.95) and ML bootstrap values (≥ 70%) are indicated along branches (BPP/ML).

Based on a BLAST search (https://blast.ncbi.nlm.nih.gov/Blast.cgi) using the LSU sequences, our isolates were identified as belonging to the genus *Acremonium*. To further determine the phylogenetic position of these strains, we performed a multi-locus phylogenetic analysis. The dataset was composed of LSU (1–430 bp) and ITS (431–1005 bp) gene, comprising a total of 1005 characters (including gaps). The best-fit partition model for ML analysis and BI analysis is shown in Table 2. The results showed that the CGMCC 3.20955, GZUIFR 22.037, and GZUIFR 22.038 are still grouped in the *Pinkertoniae*-clade (Fig. 1). The CGMCC 3.20954 is still grouped in the *Chrysogenum*-clade (Fig. 1).

**Table 2.** The best-fit substitution models are used in multi-locus phylogenetic construction.

	LSU	ITS
ML analysis	TN+F+R5	GTR+F+R4
BI analysis	GTR+F+I+G4	GTR+F+I+G4

### ﻿Taxonomy

#### ﻿ Acremonium globosisporum

Taxon classificationFungiHypocrealesBionectriaceae

Xin Li, Y.F. Han & Z.Q. Liang sp. nov.

35BF27DA-FC43-57D8-989A-49581DCE8C51

 843765

Fig. 2

##### Type.

Yuncheng East Garden Wildlife Zoo, Yuncheng City, Shanxi Province, China N35°6'26", E111°4'24", isolated from green belt soil, July 2021, Yu-Lian Ren (dried holotype culture HMAS 351939, ex-holotype culture CGMCC 3.20955 = GZUIFR 22.036). ITS sequences, GenBank ON041035; LSU sequences, GenBank ON041051; SSU sequences, GenBank ON876755; *TEF 1-α* sequences, GenBank ON494580; *RPB2* sequences, GenBank ON494584.

**Figure 2.**
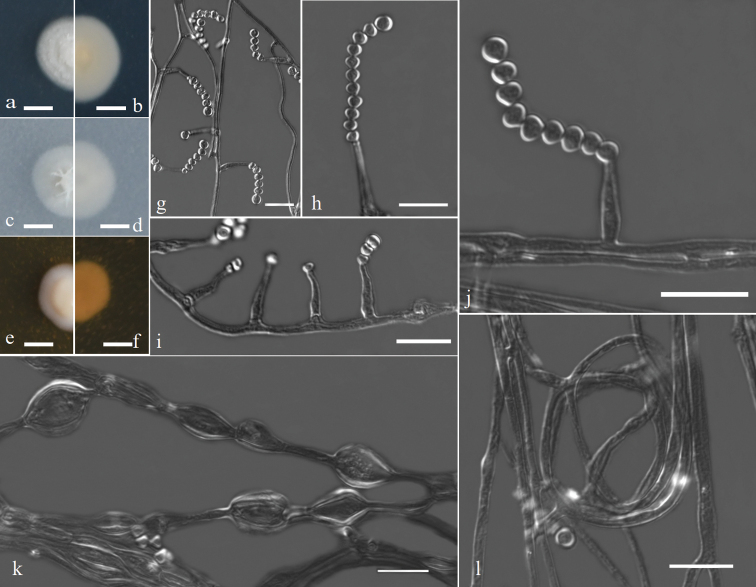
Morphology of *Acremoniumglobosisporum* sp. nov. **a–f** colony on PDA, OA and MEA after 7 d at 25 °C (upper surface and lower surface) **g–j** conidia are borne on the phialides **k–l** winding hyphae and inflate hyphae. Scale bars: 4 mm (**a–f**); 10 μm (**g–l**).

##### Description.

*Colonies* on PDA and OA at 25 °C attaining 11–13 mm and 9–11 mm diam respectively after 7 d, white, flat or raised, velvety to slightly cottony. On MEA at 25 °C, reaching 8–10 mm after 7 d, white to yellowish white, raised, slimy. *Hyphae* hyaline, septate, sometimes winding and inflate, 1.5–11.0 µm wide. *Sporulation* abundant. *Phialides* are mostly borne singly, hyaline, erect to slightly curved, sometimes forming a collarette, 9.0–22.0 µm long, tapering from 1.5–3.5 µm near the base to 0.5–1.5 µm. *Conidia* cohering in long chains, with minutely truncate ends, up to 27.5 µm long, globose or subglobose, 2.5–4.5 × 2.5–4.5 µm (x– ± SD = 3.4 ± 0.77 × 3.6 ± 0.52, n = 50) diam. *Chlamydospores* and teleomorph stage were not observed.

##### Etymology.

*globosisporum*. A reference to the global conidia.

##### Additional specimens examined.

Yuncheng East Garden Wildlife Zoo, Yuncheng City, Shanxi Province, China N35°6'26", E111°4'24", isolated from green belt soil, July 2021, Yu-Lian Ren, GZUIFR 22.037, ITS, LSU, SSU, *TEF 1-α*, *RPB2* sequences GenBank ON041036, ON041052, ON876756, ON494581, ON494585; GZUIFR 22.038, ITS, LSU, SSU, *TEF 1-α*, *RPB2* sequences GenBank ON041037, ON041053, ON876757, ON494582, ON494586.

##### Known distribution.

Yuncheng City, Shanxi Province, China.

##### Notes.

The phylogeny results showed that the CGMCC 3.20955, GZUIFR 22.037 and GZUIFR 22.038 still nested in the *Pinkertoniae*-clade. The morphological characteristics of *Acremoniumglobosisporum* were similar to other species of the *Pinkertoniae*-clade in that phialides were erect on the hyphae; sporulation was abundant, and conidia were subglobose (Ito et al. 2000). However, *Acremoniumglobosisporum* hyphae were sometimes winding and inflated, with conidia cohering in long chains, unlike other species.

#### ﻿ Acremonium curvum

Taxon classificationFungiHypocrealesBionectriaceae

Xin Li, Y.F. Han & Z.Q. Liang sp. nov.

7DC71370-B32D-59C2-B927-D407C2A66239

 843766

Fig. 3

##### Type.

Zhengzhou Zoo, Zhengzhou City, Henan Province, China N34°47'20", E113°40'41", isolated from green belt soil, July 2021, Yu-Lian Ren (dried holotype culture HMAS 351938, ex-holotype culture CGMCC 3.20954 = GZUIFR 22.035). ITS sequences, GenBank ON041034, LSU sequences, GenBank ON041050; SSU sequences, GenBank ON876754; *TEF 1-α* sequences, GenBank ON494579; *RPB2* sequences, GenBank ON494583.

**Figure 3.**
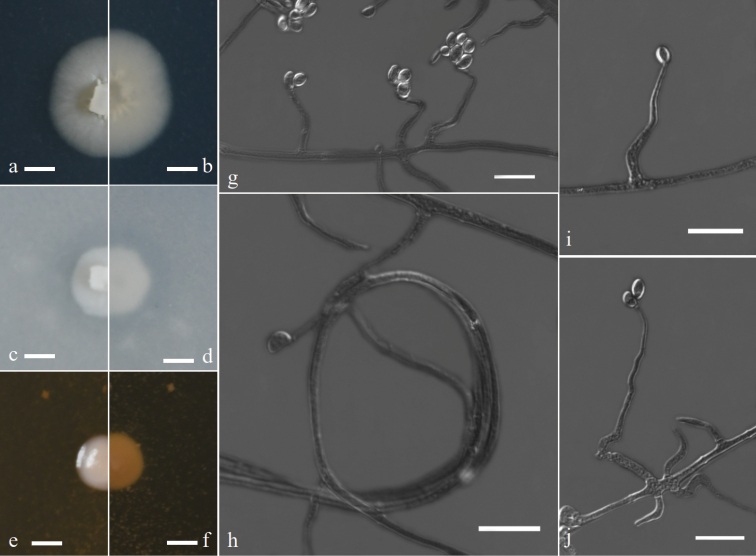
Morphology of Acremoniumcurvum sp. nov. **a–f** colony on PDA, OA and MEA after 7 d at 25 °C (upper surface and lower surface) **g, I, j** conidia are borne on the phialides **h** Winding hyphae. Scale bars: 4 mm (**a–f**); 10 μm (**g–j**).

##### Description.

*Colonies* on PDA and OA at 25 °C attaining 11–14 mm and 7–9 mm diam respectively after 7 d, white, flat, radially folded or rugose. On MEA at 25 °C, reaching 6–8 mm after 7 d, white to yellowish-white, slimy. *Hyphae* hyaline, septate, sometimes winding, 1.5–2.5 µm wide. *Sporulation* abundant. *Phialides* are mostly borne singly, curved, slightly inflated at the base, tapered at the tip, up to 38.0 µm long. tapering from 1.5–3.5 µm near the base to 0.5–1.5 µm. *Conidia* cohering together on the top of phialides, one-celled, solitary, or several fascicled, ovoid or subglobose, 3.0–7.0 × 2.5–3.5 µm (x– ± SD = 4.1 ± 1.18 ×3.2 ± 0.77, n = 50) diam. *Chlamydospores* and teleomorph stage were not observed.

##### Etymology.

*curvum*. Referring to the curved Phialides.

##### Known distribution.

Henan Province, China.

##### Notes.

Based on the multi-locus analysis we found that *Acremoniumcurvum* had close phylogenetic affinities to other taxa of the *Chrysogenum*-clade. Morphologically, *A.curvum* was similar to other taxa of the *Chrysogenum*-clade in having simple or rarely branched conidiophores, slightly inflated at the base and tapered at tip phialides, and ovoid to subglobose conidia (Yang et al. 2019). Conidia of *Hapsidosporairregularis* and *A.curvum* had several fascicled at the tips of the conidiophores (Malloch and Cain 1970). However, *A.curvum* was differentiated by having mostly curved phialides and the conidia were several fascicled at the tips of the phialides.

## ﻿Discussion

In the present study, four strains of *Acremonium* fungi were isolated from soil in the Shanxi and Henan Province, China. Two-locus (LSU and ITS) phylogenetic analyses in combination with morphological characteristics were used for identification. As a result, two new species of *A.curvum* (one isolate) and *A.globosisporum* (three isolates) were proposed.

With the development of biotechnology, a growing number of studies have combined morphological and phylogenetic features to distinguish between species. This provides the basis for more precise species naming. Generally, the fungal ITS marker includes considerably more sequence variability, and consequently provides high interspecific resolution, and also some degree of intraspecific variability (Nilsson et al. 2008). Therefore, ITS has been widely used in studies of fungal inter- and intraspecific relationships (Dai et al. 2020; Szczepańska et al. 2021). There are numerous ITS sequences stored in public databases, which are incomparable to other molecular markers (Zhang et al. 2022). In addition, according to Vu et al. (2019), combining ITS and LSU can improve the accuracy of fungal species discrimination with high generality. They think that fungi commonly present in clinical, environmental, or economically relevant communities can often be identiﬁed to species level by their ITS and LSU barcodes (Vu et al. 2019).

Although, Yang et al. (2019) used a multi-locus phylogenetic analysis in introducing the new species *Acremoniumarthrinii*, lacking loci such as SSU, *TEF 1-α* and *RPB2* (Table 1) in the isolates of *Acremonium* spp. were relatively serious, so it is not difficult to find that the strains with SSU and *TEF 1-α* in this analysis were not yet 50% or even 30% of the total number of strains. Therefore, although we sequenced these above loci in the new isolates, they were not included in the phylogenetic analysis. In the future, the phylogeny relationships of *Acremonium* members will undoubtedly vary and become clearer with the increase of the number and type of molecular used.

In recent years, *Acremonium* spp. has been reported to cause immunocompetent and immunocompromised individual diseases, such as brain abscess (Anis et al. 2021), fungal keratitis (Liu et al. 2021), fungal osteomyelitis (Jalan et al. 2021), and fungal maxillary sinusitis (Durbec et al. 2011). In the present study, all strains were isolated by a method specifically designed for the isolation of keratinophilic microbes. Therefore, more studies are necessary to confirm whether *A.curvum* and *A.globosisporum* are opportunistic infectious pathogens that infect the skin and cause skin infection, as well as their potential application in the degradation of keratin-rich matrices.

## Supplementary Material

XML Treatment for Acremonium globosisporum

XML Treatment for Acremonium curvum
